# Unexpected Spontaneous Recovery From Profound Sensorineural Hearing Loss Induced by Radiation in a Patient With a Skull Base Tumor

**DOI:** 10.7759/cureus.105290

**Published:** 2026-03-16

**Authors:** Majd Khoury, Dana Egra-Dagan, Saeed Salman, Riad Khnifies

**Affiliations:** 1 Otolaryngology - Head and Neck Surgery, Bnai Zion Medical Center, Haifa, ISR; 2 Radiation Oncology, The Joseph Fishman Oncology Center, Rambam Health Care Campus, Haifa, ISR

**Keywords:** cochlear implantation, pseudoprogression, radiation therapy, schwannoma, sensorineural hearing loss

## Abstract

Radiation-induced sensorineural hearing loss (SNHL) typically manifests as progressive, irreversible hearing deterioration. We report an unusual case of profound SNHL with remarkable spontaneous recovery following radiation therapy for jugular foramen schwannoma. A 56-year-old male developed profound right-sided SNHL two months after completing radiation therapy for jugular foramen schwannoma. Initial audiometry revealed 0% word recognition scores and profound hearing loss across all frequencies. However, audiological assessment one year post-radiation demonstrated remarkable recovery, with word recognition scores improving from 0% to 92% and substantial threshold improvements at low and mid frequencies. The patient's hearing recovery eliminated the need for cochlear implantation, and he was successfully managed with acoustic amplification.

This case demonstrates that meaningful hearing recovery can occur despite an initially poor prognosis following radiation therapy for skull base tumors. Clinicians should maintain extended observation periods and ongoing audiological and radiological monitoring before considering irreversible interventions such as cochlear implantation.

## Introduction

Sensorineural hearing loss (SNHL) is a well-documented complication of radiation therapy, resulting primarily from radiation-induced damage to the cochlea and/or the cochlear nerve, and is typically more pronounced at higher frequencies [1,2]. Previous studies have suggested that early radiation-induced SNHL may have a transient and potentially reversible component [1,2]; however, word recognition performance generally remains similar to or below pre-treatment levels over time [3].

Jugular foramen schwannoma is a rare skull base tumor typically arising from the glossopharyngeal, vagus, or spinal accessory nerves. These tumors are usually diagnosed following gradual symptom progression related to lower cranial nerve deficits [4]. Radiation therapy has emerged as a preferred alternative to surgical resection for jugular foramen schwannomas due to the significant risk of cranial nerve injury associated with surgical procedures [5].

We report a unique case of profound SNHL, defined by pure-tone thresholds exceeding 90 dB HL, developing shortly after radiation therapy for jugular foramen schwannoma, followed by recovery of word recognition from 0% to 92% at one year - a degree of improvement strikingly at odds with previously reported outcomes [3]. Among the possible explanations for this unusual course is tumor pseudoprogression - transient post-radiation tumor swelling that may compress adjacent neurovascular structures [4].

## Case presentation

A 56-year-old male was referred to our department for cochlear implant candidacy evaluation due to right-sided profound SNHL following skull base radiation therapy. A skull base schwannoma was incidentally detected in 2008 following magnetic resonance imaging (MRI) for an unrelated indication. Initial management consisted of serial MRI surveillance; however, due to progressive tumor growth, a multidisciplinary tumor board recommended therapeutic intervention with radiation therapy.

**Figure 1:**
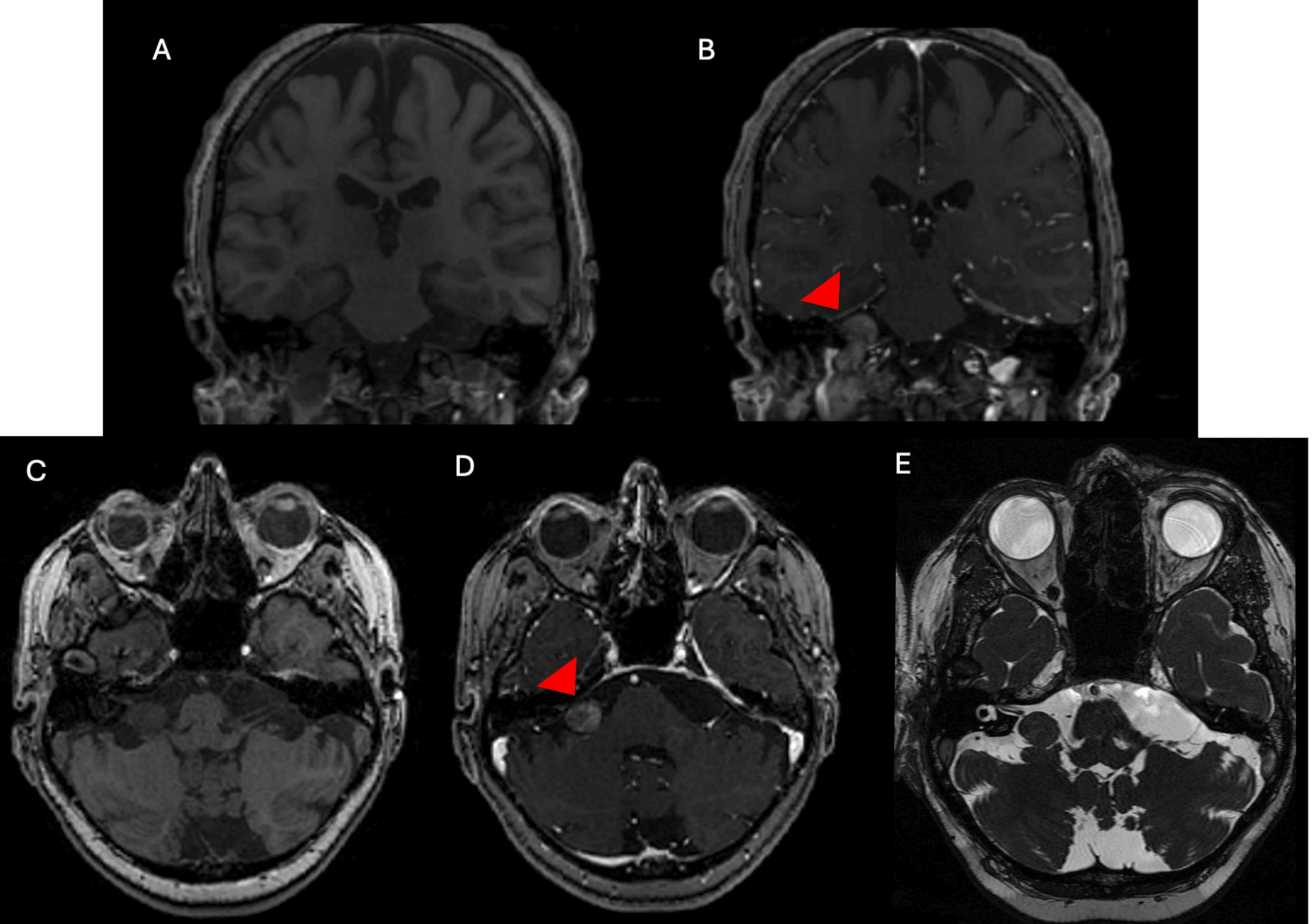
MRI characteristics of the jugular foramen schwannoma prior to radiation therapy. A dumbbell-shaped lesion centered at the jugular foramen is shown (A, B) (red arrowhead), with extension into the cerebellopontine angle cistern (C-E). The lesion demonstrates heterogeneous enhancement following gadolinium administration (B, D) (red arrowhead) and exhibits mixed signal intensity on fast imaging employing steady-state acquisition (FIESTA) sequences (E).

The last MRI performed prior to radiation demonstrated a tumor with low signal intensity on T1-weighted imaging and heterogeneous enhancement following gadolinium administration, consistent with the radiologic appearance of a schwannoma (Figure 1) [6]. The tumor extended into the prepontine cistern and mildly displaced the cochleovestibular-facial nerve complex. No contrast enhancement was evident within the internal auditory canal.

**Figure 2:**
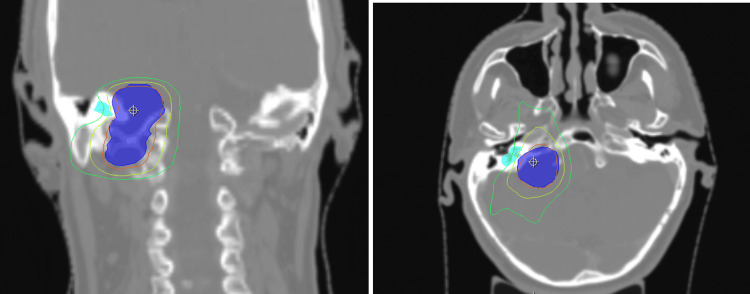
Volumetric modulated arc therapy (VMAT) treatment plan. The blue-filled volume represents the planning target volume (PTV). The cyan-filled volume indicates the right cochlea. Isodose lines are displayed as follows: red = 51.30 Gy (95% isodose line, prescription dose coverage); yellow = 40 Gy; and green = 27 Gy. The crosshair marks the treatment isocenter. PTV coverage was homogeneous, with V95 = 96% and no hotspots exceeding 105% of the prescribed dose (V105% = 0%).

A total dose of 54 Gy was administered in fractions of 2 Gy each between December 20, 2022, and January 25, 2023. Computed tomography (CT) simulation with intravenous contrast enhancement was performed and fused with a high-resolution T1-weighted MRI with a 1-mm slice thickness. Treatment was delivered using volumetric modulated arc therapy (VMAT), with immobilization achieved via a three-point thermoplastic mask (Figure 2). The mean cochlear dose remained below the recommended constraint of 45 Gy, as specified by the Quantitative Analysis of Normal Tissue Effects in the Clinic (QUANTEC) guidelines [2].

**Figure 3:**
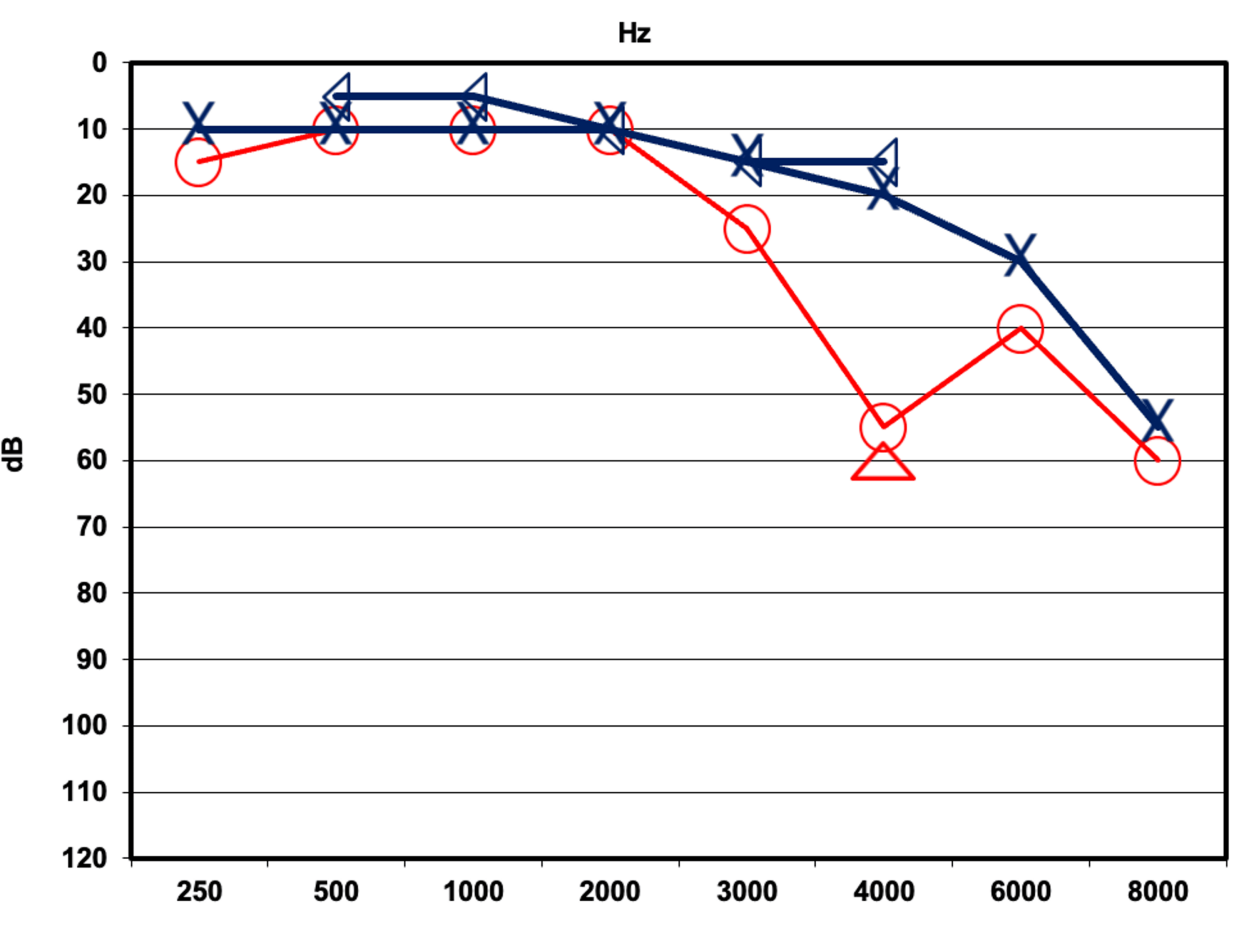
Pre-radiation pure-tone audiogram demonstrating baseline hearing thresholds. The last audiogram performed before radiotherapy showed normal hearing thresholds through 3,000 Hz in the right ear, with a decline at 4,000-8,000 Hz. The left ear demonstrated normal hearing, except for a slight-to-moderate sensorineural hearing loss (SNHL) at 4,000-8,000 Hz. Red symbols represent the right ear, and blue symbols represent the left ear. X: left-ear air conduction; ◅: left-ear bone conduction; ○: right-ear air conduction; ∆: right-ear masked air conduction SNHL: sensorineural hearing loss

His last available audiogram, obtained approximately 10 years before the initiation of radiation therapy, demonstrated asymmetric right-sided high-frequency sensorineural hearing loss (SNHL), with normal hearing thresholds preserved up to 2 kHz (Figure 3). This temporal gap limits certainty regarding the true pre-treatment audiometric status; however, the contralateral ear can serve as an internal baseline for comparison, partially mitigating this limitation.

**Figure 4:**
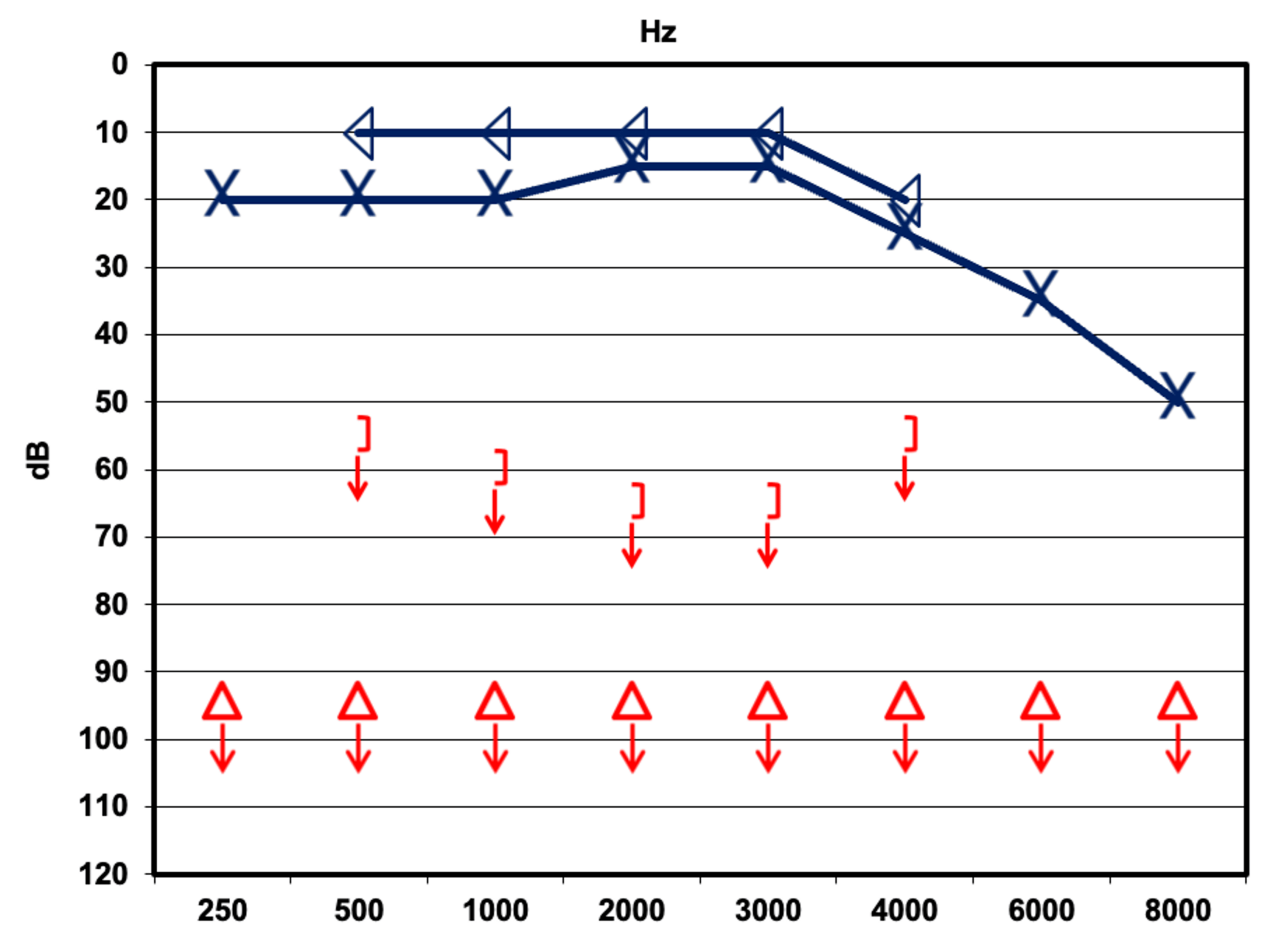
Post-radiation pure-tone audiogram at three months demonstrating profound sensorineural hearing loss. Pure-tone audiometry three months after irradiation showed profound sensorineural hearing loss involving all tested frequencies in the right ear. Red symbols represent the right ear and blue symbols represent the left ear. X: left-ear air conduction; ◅: left-ear bone conduction; ∆: right-ear masked air conduction; ]: right-ear masked bone conduction; ↓: no response at maximum output

Within two months of completing radiation therapy, the patient experienced an abrupt deterioration of hearing in the right ear, accompanied by aural fullness, tinnitus, and unsteadiness. Audiological evaluation revealed profound SNHL across all frequencies in the right ear (Figure 4). Word recognition scores were nearly 0%, consistent with profound hearing loss, indicating no usable hearing on the irradiated side. Empirical high-dose systemic corticosteroid treatment was initiated; specific details of the drug, dose, route, and duration are not available for this retrospective case report, which constitutes a limitation. No immediate audiometric benefit was observed, and a repeat audiogram five months post-radiation confirmed no recovery.

**Figure 5:**
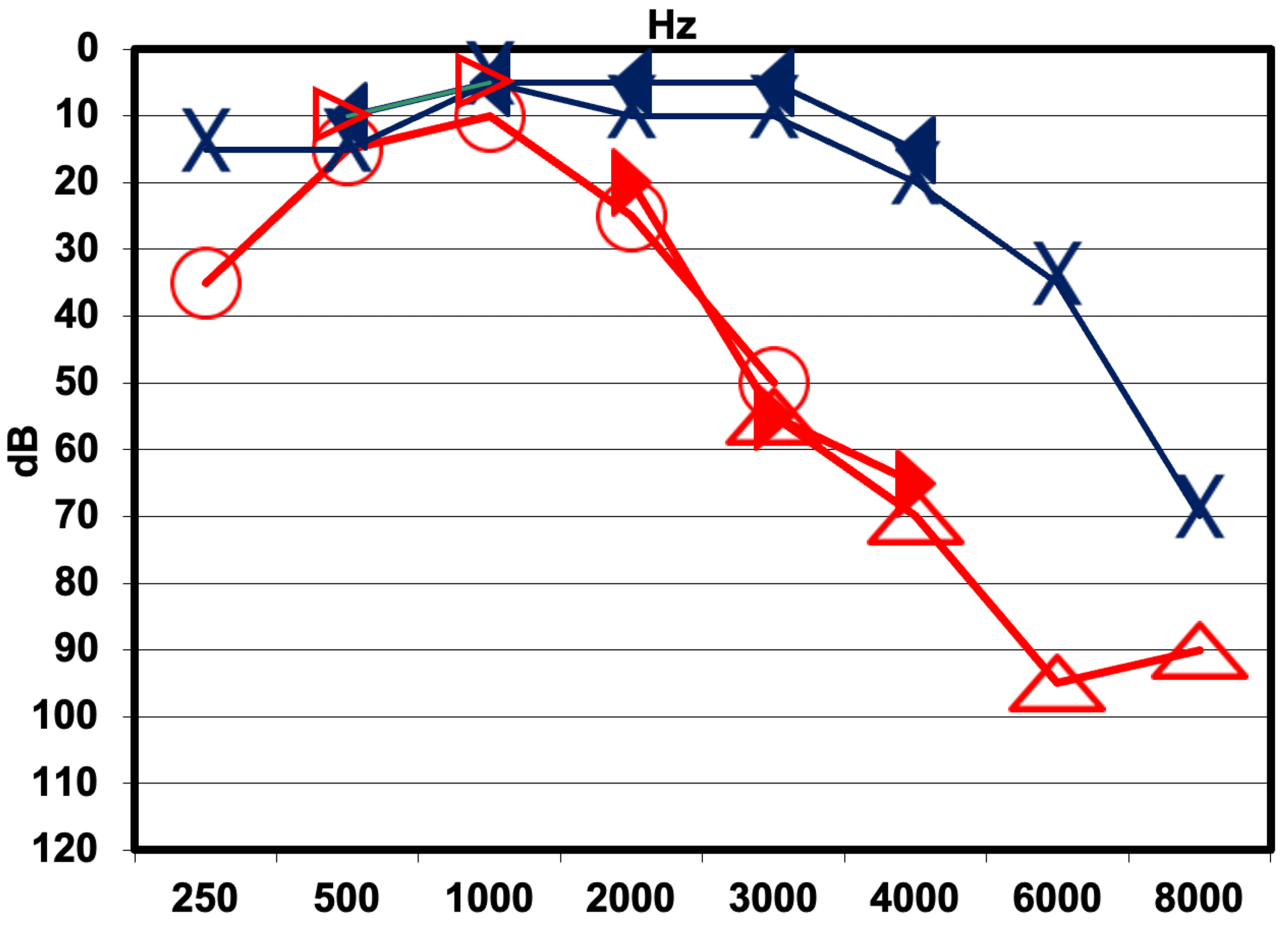
Follow-up pure-tone audiogram at one year demonstrating remarkable hearing recovery. Pure-tone bone conduction thresholds in the right ear had recovered to within normal limits from 250 Hz to 2,000 Hz, indicating restoration of low-frequency hearing sensitivity. At higher frequencies (3,000-8,000 Hz), the patient’s right ear continued to exhibit moderate-to-profound sensorineural hearing loss (SNHL), with only partial recovery. Red symbols represent the right ear, and blue symbols represent the left ear. X: left-ear air conduction; ◀: left-ear masked bone conduction; ○: right-ear air conduction; ∆: right-ear masked air conduction; ▶: right-ear masked bone conduction

An audiological assessment one year following radiation demonstrated remarkable improvement in right ear hearing, including substantial recovery in hearing thresholds, a speech reception threshold of 25 dB HL, and a word recognition score of 92%, closely approximating the pre-radiation audiometric baseline (Figure 5).

Further audiological evaluation included speech perception tests using recorded material, specifically the Hebrew versions of the Arthur Boothroyd monosyllables and AzBio adaptive signal-to-noise ratio-50 (SNR50) tests (Table 1) [7], along with the Speech, Spatial, and Qualities of Hearing (SSQ12) questionnaire (Table 2) [8]. These findings are consistent with the marked improvement in hearing thresholds and word recognition scores observed on behavioral audiometric testing.

**Table 1:** Speech perception test results. Results of HAB monosyllables and Hebrew AzBio (He-AzBio) adaptive SNR50 tests under different listening conditions. He-AzBio: Hebrew AzBio; HAB: Hebrew Arthur Boothroyd monosyllables; BAHA: bone-anchored hearing aid; SNR: signal-to-noise ratio

Test condition	Unaided	+BAHA	BAHA only
HAB in quiet	100%	-	80%
HAB in 4-babble talker SNR +0	25%	20%	0%
He-AzBio (SNR50)	2.8 dB	3.8 dB	9.6 dB

**Table 2:** Speech, Spatial and Qualities of Hearing (SSQ12) scale results. Comparison of patient scores with bimodal hearing aid users. Scores range from 0 (worst) to 10 (best). Bimodal group data represent normative values for patients using bilateral hearing aids (and not a matched control).

Variables	Speech in noise	Spatial hearing	Qualities of hearing	Total score
Our patient	5.5	3	4	4.3
Bimodal group	5.41	4.1	6.52	5.34

Given the patient's recovery of usable hearing at low and mid frequencies, combined with excellent word recognition, he was no longer considered a candidate for cochlear implantation. Instead, audiological rehabilitation focused on acoustic amplification strategies. A high-power, open-fit hearing aid, optionally combined with a contralateral routing of signal (CROS) device, was recommended for the right ear to enhance residual hearing, particularly addressing high-frequency deficits. Assistive listening devices and communication strategies were also advised. At the last follow-up, the patient reported improved quality of life and successful communication during one-on-one conversations with the hearing aid.

## Discussion

This case illustrates an uncommon but important phenomenon of reversible profound SNHL following radiation therapy for skull base tumors. While large clinical series have shown that radiation-induced SNHL typically manifests as a delayed, progressive decline in hearing with limited potential for improvement, the present case demonstrates a markedly different clinical course [1,3]. Radiation-susceptible structures within the temporal bone include the cochlea, particularly its basal turn, the modiolus, and the internal auditory canal, each demonstrating distinct audiological consequences upon exposure [3].

In this patient, a global deterioration in auditory thresholds and word recognition scores occurred two months following treatment completion, suggesting injury to the retrocochlear auditory pathway [3]. However, subsequent audiometric assessment at one year demonstrated significant improvement, predominantly at low and mid frequencies, with word recognition scores recovering from 0% to 92%.

The mechanism underlying this remarkable recovery remains unclear, though several possibilities warrant consideration. One potential explanation is transient tumor expansion following radiation therapy, a phenomenon known as pseudoprogression, which could temporarily compress the cochlear nerve before subsequently resolving [4,9]. This process has been documented in both vestibular schwannomas and jugular foramen schwannomas, with Kim et al. observing transient tumor expansion in approximately one-third of jugular foramen schwannoma patients following radiation therapy [4]. While the exact pathophysiology remains speculative, the temporal pattern observed in our patient, acute hearing loss within two months followed by recovery at one year, aligns with documented pseudoprogression timelines [9]. Although Aoyama et al. found that hearing typically does not recover after tumor shrinkage [10], the present case suggests that recovery is possible, particularly when edema-related effects respond to anti-inflammatory processes over time [4,11].

Persistent elevated thresholds at high frequencies (≥3 kHz), despite improvement at lower frequencies, suggest an additional injury mechanism. This audiometric pattern, characterized by high-frequency hearing loss with preserved speech discrimination, indicates damage predominantly involving the basal turn of the cochlea, resembling sensory presbycusis [3]. Outer hair cells (OHCs) at the basal cochlear turn are particularly susceptible to radiation-induced oxidative stress due to lower antioxidant glutathione reserves compared with apical OHCs [12]. Therefore, we propose that two distinct injury mechanisms may have contributed to this pattern. Auditory brainstem response (ABR) and otoacoustic emission (OAE) testing, which would have provided site-of-lesion information distinguishing cochlear from retrocochlear injury, were not performed - a methodological limitation of this report.

Notably, most hearing-outcome data cited above derive from stereotactic radiosurgery (SRS), whereas our patient received conventionally fractionated radiation therapy [1,3,4,9]. Whether the radiobiological advantages of lower dose-per-fraction, including greater sublethal damage repair and reduced microvascular injury, contributed to the favorable recovery cannot be determined from this case alone [12].

**Figure 6:**
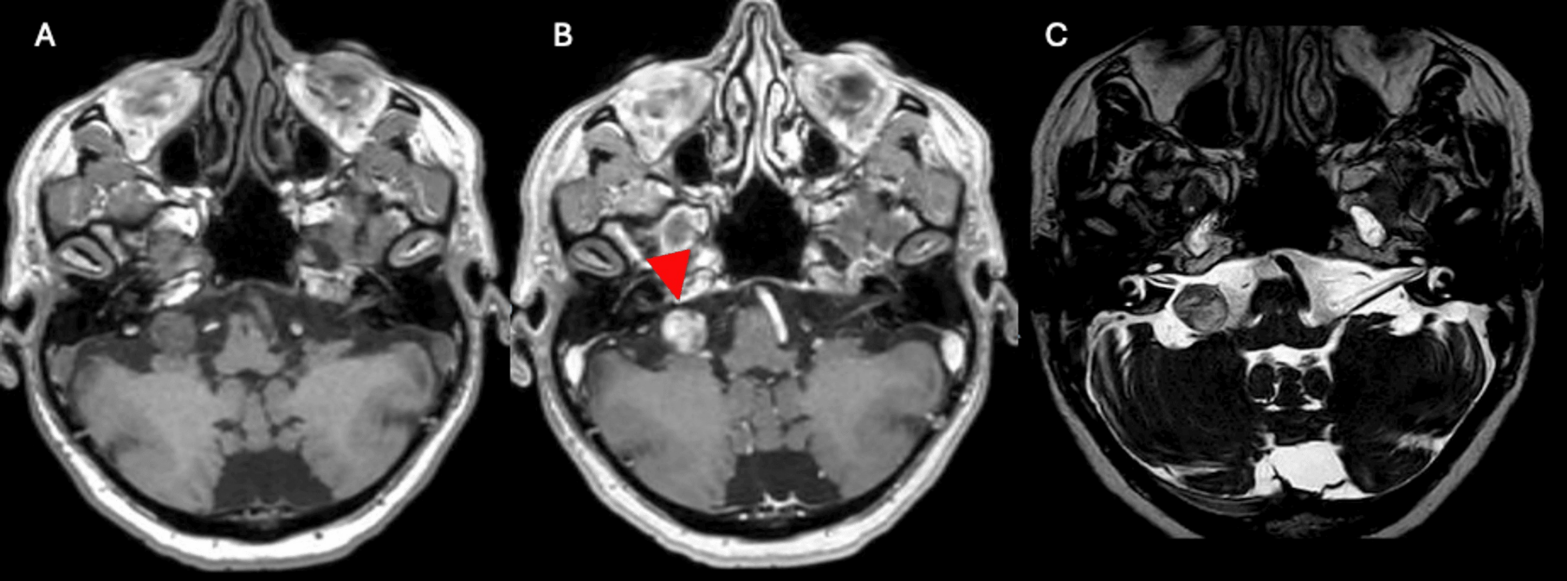
MRI characteristics of the jugular foramen schwannoma after radiation therapy. The lesion is shown on T1-weighted imaging without contrast (A) and with gadolinium administration (B), demonstrating heterogeneous enhancement (B) (red arrowhead). Compared to pre-treatment imaging, gadolinium enhancement appears more pronounced, though this finding should be interpreted with caution. Fast imaging employing steady-state acquisition (FIESTA) sequence (C) reveals a hyperintense signal at the lesion site, which also appears more conspicuous relative to prior imaging.

This case underscores the importance of ongoing audiological monitoring in patients with post-radiation hearing loss from skull base tumors. Clinicians should recognize that meaningful recovery can occur despite a poor initial prognosis, influencing treatment decisions and patient counseling. Serial MRI examinations may help identify potential causes of acute hearing changes, such as tumor-related effects. No MRI was obtained during the acute hearing deterioration phase; however, an MRI acquired at the time of recovery demonstrated changes in the tumor enhancement pattern (Figure 6). Whether these findings represent pseudoprogression remains uncertain, as no clear diagnostic imaging criteria for pseudoprogression have been established in the literature [13]. Given the potential for delayed improvement, extended observation periods should be considered before irreversible interventions such as cochlear implantation.

## Conclusions

Profound radiation-induced sensorineural hearing loss following skull base tumor treatment can undergo clinically meaningful spontaneous recovery. These findings highlight the importance of audiological follow-up for at least 12 months following radiation before considering irreversible interventions such as cochlear implantation, as recovery may continue well beyond the acute post-treatment period.
